# Supplementary material for: Biopsy Confirmed Glioma Recurrence Predicted by Multi-Modal Neuroimaging Metrics
Source: J Clin Med. 2019 Aug 23;8(9):1287. doi: 10.3390/jcm8091287 (PMC6780506; doi:10.3390/jcm8091287)
Supplement: Supplementary file 1 [file jcm-08-01287-s001.zip › Figure S1.pdf]

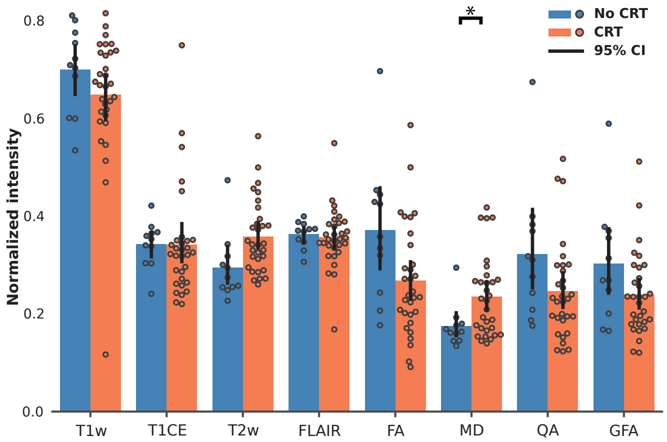

Figure S1 – Comparing average signal intensities from *Normal* ROIs between treatment groups. Patients who received CRT (orange) and those that did not (No CRT) have equal average intensities in all MR modalities investigated aside from MD. Error bars indicate 95% confidence intervals.
